# Diagnostic Dilemmas for Underlying Pathophysiology of Arrhythmias Originating from the Right Ventricle

**DOI:** 10.1016/s0972-6292(16)30737-9

**Published:** 2014-03-12

**Authors:** Serkan Cay, Nurdan Cay, Ugur Canpolat, Ozcan Ozeke, Baris Sensoy

**Affiliations:** 1 Department of Cardiology, Yuksek Ihtisas Heart-Education and Research Hospital, Ankara, Turkey; 2 Department of Radiology, Yildirim Beyazit University, Faculty of Medicine, Ataturk Education and Research Hospital, Ankara, Turkey

**Keywords:** Right Ventricular Tachycardia, Left Ventricular Non-compaction Cardiomyopathy

We have read with great interest the case presentation entitled 'Successful Right Ventricular Tachycardia Ablation in a Patient with Left Ventricular Non-compaction Cardiomyopathy' by Honarbakhsh et al. in the recent issue of the journal [1]. They successfully ablated an arrhythmia focus originated from the normally appearing right ventricular free wall using the three-dimensional mapping and remote magnetic navigation in a patient with left ventricular noncompaction cardiomyopathy (NCC). The authors have stated that anatomical structure of the right ventricular free wall containing the arrhythmia focus was normal on steady-state free precession cines and late gadolinium enhancement images. An important thing that should be considered for this type of case is the possible involvement of the right ventricular myocardium by fibro-fatty infiltration called 'arrhythmogenic right ventricular dysplasia/cardiomyopathy (ARVD/C)'. Patients with ARVD/C might be presented with ventricular arrhythmias as the first manifestation of the disease. Although the majority of patients with ARVD/C having life-threatening ventricular arrhythmias have an abnormal baseline ECG, a nonspecific or normal appearing ECG does not preclude ARVD/C diagnosis [2]. Current gold-standard non-invasive additional modality to other cardiac tests such as conventional/signal-averaged ECG and ambulatory Holter monitoring for the diagnosis and evaluation of patients with ARVD/C seems to be cardiac magnetic resonance (CMR) imaging. CMR can show the right ventricle in three and/or four dimensional format using multi-planar views. It has also very high spatial and temporal resolution resulting in highly improved contrast between blood and myocardium allowing the assessment of ventricular regional and global diastolic/systolic functions. CMR can also specifically delineate intra-myocardial fatty infiltration and replacement fibrosis which are observed as areas of high signal intensity on T1-weighted and late gadolinium images, respectively [3]. Adding fat-suppressed sequences to conventional fast spin-echo imaging allows delineation of even subtle degrees of intra myocardial fatty infiltration [4]. Therefore, early diagnosis and close-follow-up of patients can be possible although infiltrative pattern on CMR is not a criterion for diagnosis [5]. The authors of the case have mentioned only about NCC although NCC and ARVD/C might be seen in the same patient [6]. Different arrhythmia foci from different parts of the right ventricle should raise the suspicion of ARVD/C such as in the presented case. Because the patient had a history of previous ablation of ventricular arrhythmia originated from the right ventricular outflow tract.

In conclusion, differential diagnosis of ARVD/C should be kept in mind in patients with multiple arrhythmia foci from different parts of the right ventricular myocardium.
